# The Effect of a 12-Week Beta-hydroxy-beta-methylbutyrate (HMB) Supplementation on Highly-Trained Combat Sports Athletes: A Randomised, Double-Blind, Placebo-Controlled Crossover Study

**DOI:** 10.3390/nu9070753

**Published:** 2017-07-14

**Authors:** Krzysztof Durkalec-Michalski, Jan Jeszka, Tomasz Podgórski

**Affiliations:** 1 Department of Hygiene and Human Nutrition, Poznan University of Life Sciences, 60-624 Poznań, Poland; jeszkaj@up.poznan.pl; 2 Polish Wrestling Federation, 00-871 Warsaw, Poland; 3 Department of Biochemistry, Poznan University of Physical Education, 61-871 Poznań, Poland; podgorski@awf.poznan.pl

**Keywords:** β-hydroxy-β-methylbutyric acid, body composition, maximal oxygen uptake, muscle power indices, supplements, sport, training support, adaptation

## Abstract

The aim of this study was to verify the effect of beta-hydroxy-beta-methylbutyrate (HMB) supplementation on physical capacity, body composition and the value of biochemical parameters in highly-trained combat sports athletes. Forty-two males highly-trained in combat sports were subjected to 12 weeks of supplementation with HMB and a placebo in a randomized, placebo controlled, double-blind crossover manner. Over the course of the experiment, aerobic and anaerobic capacity was determined, while analyses were conducted on body composition and levels of creatine kinase, lactate dehydrogenase, testosterone, cortisol and lactate. Following HMB supplementation, fat-free mass increased (*p* = 0.049) with a simultaneous reduction of fat mass (*p* = 0.016) in comparison to placebo. In turn, after HMB supplementation, the following indicators increased significantly in comparison to the placebo: the time to reach ventilatory threshold (*p* < 0.0001), threshold load (*p* = 0.017) and the threshold HR (*p* < 0.0001), as well as anaerobic peak power (*p* = 0.005), average power (*p* = 0.029), maximum speed (*p* < 0.001) and post-exercise lactate concentrations (*p* < 0.0001). However, when compared to the placebo, no differences were observed in blood marker levels. The results indicate that supplying HMB promotes advantageous changes in body composition and stimulates an increase in aerobic and anaerobic capacity in combat sports athletes.

## 1. Introduction

β-hydroxy-β-methylbutyric acid (HMB) is a metabolite of leucine and 2-ketoisocaproic acid. For almost twenty years, it has attracted special attention as an interesting supplementation support in sports [1,2,3,4,5,6,7,8]. A major advantage of its use suggested in the literature is connected with its anti-catabolic action, manifested particularly in the following contexts: when an athlete needs to carry a heavy load, when muscle damage is experienced [2,4,9,10], and when there has been body mass loss, muscle mass loss, or some degree of cancerous cachexia [11,12,13]. It also resulted in improvement of protein metabolism and muscle work capacity in rheumatoid cachexia [14], muscular dystrophy [15], sarcopenia [10,16] or in patients who are bedridden, after injuries, suffer from lung diseases or HIV infection [17,18,19,20,21,22].

These beneficial effects might be connected with the anti-proteolytic effect of HMB based on the decrease of tumor necrosis factor receptor 1 (TNFR1), tumor necrosis factor-α (TNF-α), angiotensin II and interleukin-6 expression [15,23,24], and downregulation of autophagic-lysosomal and ubiquitin-proteasome system activity through normalizing Akt/FoxO axis, expression of MurF1, Atrogin-1 and proteolysis-inducing factor [10,13,25,26,27,28]. The observed advantages of HMB supplementation can be also associated with stimulation of mRNA translation, myogenic cell proliferation and protein synthesis via MAPK/ERK and PI3K/Akt pathways, stimulation of mTOR phosphorylation (which would activate the ribosomal p70^S6k^ kinase and initiation factor 4E-BP1) [9,11,23,29], expression of insulin-like growth factor 1 (IGF-1) [7,29,30,31] and growth hormone (GH) [7,30,31]. It should be noted here that the oldest reported potential influence of HMB was on cell membrane integrity through cytosol HMB’s conversion to cytosolic β-hydroxy-β-methylglutaryl-CoA (HMG-CoA), which can then be directed for mevalonate and de novo cholesterol synthesis [2,4,10,32]. Moreover, HMB may participate in the activation of AMPK kinase and Sirt 1, and promote mitochondrial biogenesis stimulation, cause higher oxygen consumption, increased efficiency of carbohydrate and fat metabolism, as well as increased lipolysis and fat mass reduction [33,34].

The advantageous effects of HMB supplementation in physically active individuals mainly consisted in changes in fat free mass or fat mass [2,3,6,8,35,36,37,38], strength [2,3,35,36,37,38], power [3,6,36,38], muscle damage (proved in the analysis of creatine kinase (CK) activity or 3-methylhistidine (3MH) levels) [2,35,36,38,39], delayed onset muscle soreness (DOMS) [39], and hormonal profiles (testosterone or cortisol levels) [7,36,38] mainly during resistance training. Some favorable changes caused by HMB supplementation were also registered in the elderly [17,40]. These studies were performed both in untrained [2,35,36,39] and trained [3,37,38] subjects, which may have influenced the obtained results (among others, due to differences in sensitivity to the exercise stimulus). A meta-analysis by Nissen and Sharp [41] demonstrated that in the case of resistance training, HMB supplementation results in increased strength and fat-free mass of net value 1.4% and 0.28% a week respectively in the case of both trained and untrained individuals. However, certain studies did not show the effect of HMB on participants involved in resistance training. No significant changes were found in muscle strength [35,42,43], power [44,45] and muscle work capacity [42], fat free mass [42], fat mass [42,43], muscle damage markers (CK) in blood [42,44,46], as well as testosterone or cortisol concentrations in blood [3,44,46].

In contrast, the influence of HMB supplementation on aerobic capacity in endurance training has rarely been verified. Only 7 papers have analyzed this aspect and they included studies of both untrained or recreationally active subjects [47,48,49,50] and elite athletes [6,8,51]. In highly trained rowers, $\dot{V}$O_2_max and ventilatory threshold increased and fat mass decreased significantly in comparison to the placebo treatment after 12 weeks of HMB supplementation [6]. Moreover, in relation to the initial values, HMB supplementation increased the time to exhaustion (Tref) in the progressive test and anaerobic peak power in Wingate test. An increase in peak oxygen uptake ($\dot{V}$O_2_peak), an extension of the time needed to reach $\dot{V}$O_2_peak and a delay in the onset of blood lactate accumulation (OBLA) were observed in HMB-supplemented trained cyclists [51]. The above observations were also confirmed by Robinson et al. [49], who reported an increase in both $\dot{V}$O_2_peak and VT after HMB supplementation in comparison to the placebo treatment. Moreover, an increase in maximal oxygen uptake ($\dot{V}$O_2_max) was also found in a group of students supplemented with HMB after a period of treadmill training [49]. In turn, Knitter et al. [47] administered HMB to a group of runners and demonstrated a lower activity of creatine kinase (CK) and lactate dehydrogenase (LDH) after the completion of a 20-km run. There is also little evidence that HMB supplementation enhances aerobic capacity. In a group of volleyball players and long-distance runners treated with HMB, no significant changes were found in maximal oxygen uptake [3,47]. Moreover, some studies of HMB-supplemented groups practicing mainly endurance exercises did not register significant changes in lean body mass [48], fat mass [47,48], CK activity [6,52] or selected hormonal marker concentrations (testosterone and cortisol) [6]. Furthermore, it should be highlighted that so far there have been limited studies involving different training procedures, e.g., those typical of combat sports or intense military training. In the case of energy-restricted female Judo athletes [45] and male soldiers from an elite combat unit [5], a greater decrease in body weight and body fat levels, attenuation of the inflammatory response and maintenance of muscle quality were, respectively, observed after HMB supplementation.

In view of the inconclusive character of the results of the studies conducted to date and of a relatively low number of studies investigating the effectiveness of HMB supplementation over a longer period on a large population of trained athletes, the aim of this study was to verify the effect of HMB supplementation on body composition, aerobic and anaerobic capacity, and on the levels of biochemical markers in highly-trained combat sports athletes.

## 2. Materials and Methods

### 2.1. Subjects

**Figure 1:**
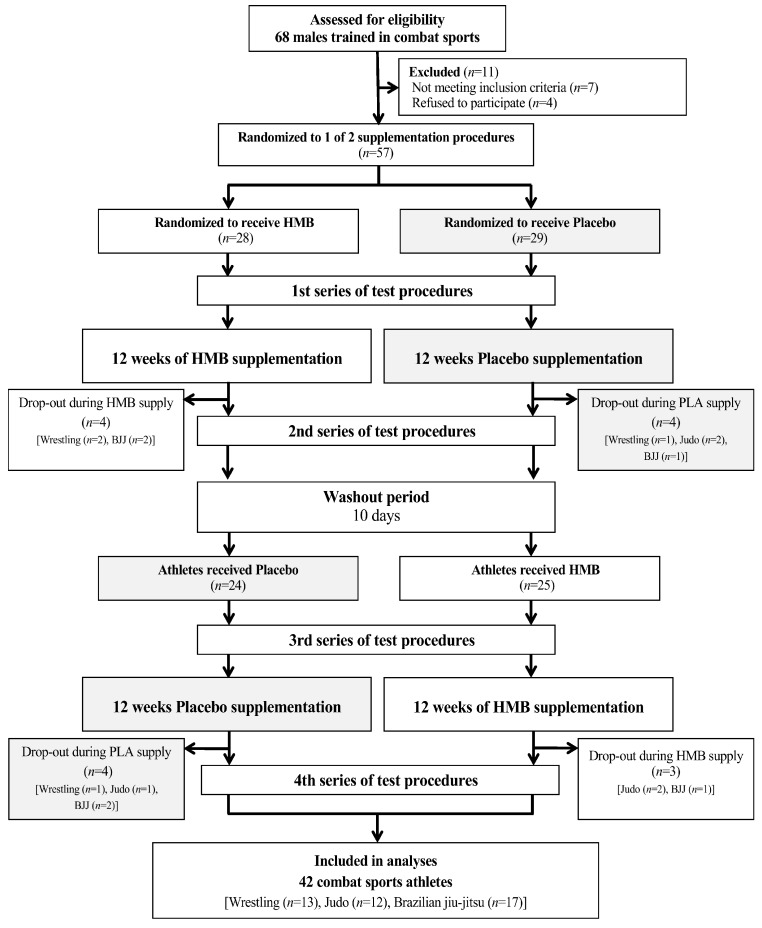
A flow chart of the study design. Abbreviations: BJJ, Brazilian jiu-jitsu; HMB, β-hydroxy-β-methylbutyric acid; PLA, placebo.

Fifty-seven males trained in combat sports were enrolled into the study, of whom 42 completed the full study protocol (13 wrestlers, 12 judokas and 17 Brazilian jiu-jitsu athletes) (Figure 1). The characteristics of the examined study group are given in Table 1. Athletes were aged 22.8 ± 6.1, with body weight of 81.2 ± 12.8 kg, height of 179 ± 6 cm, and with combat sports training experience of 7.3 ± 3.7 years. Subjects trained mostly in sports clubs in the Wielkopolska region (Poland). It was a thought-through decision that trained athletes of different combat sports disciplines were involved in this study. This was done to exclude the potential bias connected to specificity of particular training procedures. The criteria for qualifying for the study included, among others, good health condition, a valid medical certificate confirming the athlete’s ability to practice sports, at least 5 years of training experience, and a minimum of 6 workout sessions a week (minimum 3 in the practiced combat sports discipline). The studies were conducted from 2013 to 2014 at different times of year. All athletes declared that they had not introduced any changes in their lifestyles, elements of training, nutrition or supplementation, and that they had not been using any medications and supplements with potential ergogenic effects, other than those supplied by the authors of this study. Moreover, the dietary and workout records were performed every second week, which proved that athletes did not change their dietary habits and training specificity during the supplementation period (Table 1).

The study was approved by the local ethical committee (Bioethics Committee at Poznan University of Medical Sciences, Poznan, Poland. Decision No. 584/09 of 18 June 2009, **Clinical Trial Identification Number:** NCT03028649.) and written informed consent was obtained from all participants before the study began. Informed consents to the participation in the study of two athletes under the age of 18 were also obtained from their parents. All procedures were conducted in accordance with the ethical standards of the 1975 Helsinki Declaration. Trial study protocol was included as Supplementary Materials (File S1).

This trial was registered at Clinical Trials Gov (website: https://clinicaltrials.gov/ct2/show/NCT03028649?term=HMB&rank=16; Clinical Trial Identification Number: NCT03028649). The study was registered retrospectively since the registration was not required when the study enrolment started. The authors confirm that all ongoing and related trials for this intervention are registered. The study complies with the CONSORT Statement for randomized trials as shown in Figure 1 and Table S1.

### 2.2. Experimental Design

#### 2.2.1. Supplementation Characteristics

The effect of HMB supplementation was assessed in randomized crossover double-blind tests (Figure 1). Upon being qualified to the experiment, the athletes were subjected to a randomization procedure (based on their lean body mass) and assigned either to the group receiving an HMB preparation or to the group receiving the placebo. The random allocation sequence and assigned participants to supplementation of preparation with specific codes was performed by an impartial biostatistics, as well as participants were enrolled by the authors of this study. The experimental procedure for each athlete included a 12-week HMB supplementation and a 12-week placebo administration. Between the 12-week HMB and PLA or a PLA and HMB treatment, a 10-day washout period was introduced, similarly to the other studies [6,51]. It seemed to be sufficient, taking into the consideration the kinetics of HMB excretion from the body [53]. The next step, following the washout period, was a crossover exchange of the preparations administered to the groups.

The experiments were conducted using a preparation of calcium salt of β-hydroxy-β-methylbutyric acid produced by Olimp Laboratories (Dębica, Poland), that have experience in making HMB and high quality standards of production. A single capsule contained 1250 mg Ca-HMB, which corresponds to 1000 mg of β-hydroxy-β-methylbutyrate. The producer also prepared a placebo preparation containing maltodextrin. Preparations were blinded using special codes, making it impossible to identify and assign the same preparation twice to the same subject. The tested group of athletes was administered 3 capsules of the assigned preparation a day in 3 doses: upon waking, immediately after training, and before sleep. On non-training days, the participants were instructed to consume one serving with each of three separate meals throughout the day. The consumed HMB dose was equivalent to the most commonly recommended uptake of 3 g of HMB a day [2,3,4,35,48]. To ensure compliance, participants completed supplement logs, met with a professional dietician and handed in empty supplement packets.

Among all the participants, the effectiveness of HMB supplementation was assessed using four series of tests (each included an evaluation of body composition, aerobic and anaerobic capacity, blood sampling and biochemical analyses), consisting of identical procedures in two cycles separated by the washout period. The tests took place before the supplementation started (HMB_pre_ and PLA_pre_) and after the 12 weeks of supplementation with the HMB preparation (HMB_post_) and the placebo (PLA_post_) (Figure 1). The subjects did not need to be familiarized with the tests because they were acquainted with the Wingate test and the incremental test with increasing intensity used in some previous studies and training procedures. As mentioned in the Methods Section, all the athletes declared that they had not undergone changes in their lifestyles, elements of training or nutrition.

#### 2.2.2. Anthropometry and Body Composition

Body mass and height were measured using a WPT 60/150 OW medical anthropometer by RADWAG^®^ (Radom, Poland). Body composition was analyzed by determining the values of electrical resistance and reactance through bioelectric impedance with the use of a BIA 101S analyzer by AKERN-RJL (Pontassieve, Italy) and Bodygram 1.31 computer software by AKERN-RJL (Pontassieve, Italy). Body composition was measured strictly following the recommended measurement conditions: In the morning hours following overnight fasting, while lying in a supine position, and with the recommended measuring electrode application [54]. Athletes were also instructed to abstain from drinking coffee, strong tea, caffeine-containing products and alcohol for at least 24 h before the test, and to refrain from physical exercise for a minimum of 18 h before the measurements. The coefficient of variation (CV) for the analysis of total body fat was less than 3.0% (2.1–2.9%), similar to the results reported by Erceg et al. [55] and Kyle et al. [54]. Furthermore, following the idea adopted from Kraemer et al. [36], before each of the four series of tests, the hydration status of athletes was verified by urine specific gravity measurement conducted with a handheld refractometer and results of <1.020 were recognized as indicator of proper hydration.

#### 2.2.3. Aerobic Capacity

Exercise tests to assess the selected capacity parameters were conducted in the morning hours (between 7:00 and 10:30 a.m.), always in the same conditions (temperature of 20–22 °C, relative humidity of 50–60%). Prior to each test, the athletes were informed in detail about the objective, procedure and tests methods. The level of aerobic capacity was assessed based on the recorded maximal oxygen uptake ($\dot{V}$O_2_max) and the ventilatory threshold (VT) during a test that involved performing exercise with an increasing intensity (+50 W each 3 min) on Kettler X1 cycloergometer (Kettler, Ense-Parsit, Germany), following the recommendations for such tests [56]. During the tests, respiration indices were recorded using a portable K4b^2^ ergospirometer (Cosmed, Rome, Italy) and COSMED CPET Software Suite (ver. 9.1b, 2010). Moreover, Cosmed K4b^2^ system was calibrated prior to each individual test in accordance with the manufacturer’s guidelines.

In this study, maximal exercise was assumed to occur when an increase in load failed to produce an increase in oxygen uptake ($\dot{V}$O_2_) and heart rate (HR). To determine the ventilatory threshold (VT), the V-slope method was applied based on an analysis of the linear regression for the curve of increasing CO_2_ exhalation in comparison to the curve of increasing O_2_ uptake [57].

#### 2.2.4. Anaerobic Capacity

Anaerobic capacity was assessed using the classical Wingate test on a cycloergometer (Monark 894E, Varberg, Sweden), following the recommendations for such tests proposed by Bar-Or [58]. The seat height was adjusted to each participant’s satisfaction, and toe clips with straps were used to prevent feet from slipping off the pedals. The primary test was preceded by a 5-min warm-up period of approximately 50 W power, with a 5-min break afterwards. This was followed by two run-up practices of 3 s, during which the actual test load was imposed to make the participants accustomed to the resistance. The test lasted for 30 s. External loading was estimated individually at 7.5% body weight. During the test, the athletes were encouraged to put in maximum effort. The recorded results included the peak power output, the average power output, the minimal power output, and the maximum speed, which were analyzed using Monark Anaerobic Test Software (ver. 3.0.1, 2009, Varberg, Sweden).

#### 2.2.5. Blood Sampling and Biochemical Analyses

This investigation applied markers of adaptation and homeostasis the most widely used in studies involving athletes. The activity of the creatine kinase and lactate dehydrogenase enzymes and the concentrations of the hormones (testosterone and cortisol) and lactate were assessed based on a quantitative analysis of the blood plasma of the athletes using commercial diagnostic tests. In numerous studies concerning HMB, its amount in blood was checked before exercises, in fasting state, and/or early in the morning [2,3,35,36,42,44,46,47,49], and immediately after exercising [36,47,59]. In our opinion, in combat sport practice, it is important to evaluate the physiological response not only immediately after exercising, but also a few minutes later, especially when athletes make an effort in a relatively short recovery periods (this concerns mainly combat sports). For this reason, the authors were interested in examining whether the HMB supplementation affects muscle damage enzyme activity, and lactate, testosterone or cortisol concentration in blood exactly 20–25 min after a maximal aerobic incremental capacity test. Blood samples were collected from the athletes’ ulnar vein to two closed-system evacuated test tubes of 2.7 mL, using lithium heparine and sodium fluoride as anticoagulants (Sarstedt Monovette^®^, Sarstedt, Germany). The collected plasma was subjected to further laboratory analyses on the same day. Creatine kinase (CK) and lactate dehydrogenase (LDH) activity, as well as the concentration of lactate, were assayed using a standardized colorimetric enzymatic method with a COBAS^®^ 6000 analyzer (module c 501, Roche/Hitachi, Indianapolis, IN, USA). The concentrations of testosterone and cortisol in blood plasma were assayed by ECLIA electrochemiluminescence using a COBAS^®^ 6000 analyzer (module e 601, Roche/Hitachi, Indianapolis, IN, USA). Moreover, at rest and three minutes after the Wingate anaerobic test, lactate concentration in capillary blood was determined using the spectrophotometric enzymatic method [59].

#### 2.2.6. Statistical Analysis

The study was powered based on our previous study [6] to detect a difference in anaerobic peak power between HMB and placebo treatment. A 2-sided *t*-test with 30 individuals in a crossover design would achieve 80% power to detect a 2.94% difference in the mean, assuming 15.87% standard deviation of the difference and alpha = 0.05. All statistical calculations were performed using Statistica 12.0 package (StatSoft, 2014, license No. JPZP512B037809AR-3). Basic descriptive statistics were calculated for the tested parameters, and the results are presented here as arithmetic means and standard deviations (±SD) for at least four independent series of measurements. The Shapiro–Wilk test was applied in order to determine whether the random sample came from a population with a normal distribution. The statistical analysis was performed based on the research hypothesis that HMB supplementation supports physical capacity and body composition regulation in trained combat sports athletes. Therefore, statistical tests were selected in order to compare the significance of the changes resulting from HMB supplementation or placebo administration. Since a crossover design was used in this study and all subjects received both HMB and placebo, the significance of the differences in changes after HMB-supplemented and placebo treatment subjects, as well as the differences in mean values of parameters between the baseline (Pre) and post-intervention (Post) results (within each group—HMB and PLA) were tested by paired 2-tailed dependent sample *t*-tests (normally distributed variables) or Wilcoxon-signed rank tests (non-normally distributed variables). In turn, the significance of the differences in the mean value of parameters between “Pre” (HMB vs. PLA), and “Post” (HMB vs. PLA) were tested using 2-tailed independent samples *t*-tests in the case of normally distributed variables, or with Mann–Whitney U tests in the case of non-normally distributed variables. In addition, to verify whether the order of HMB or placebo administration affected the recorded results, ANOVA for repeated measurement with Time (Pre-Post), Treatment (HMB vs. PLA) and Supplementation Order (HMB➔PLA vs. PLA➔HMB) was also performed in this study.

## 3. Results

### 3.1. Body Composition

**Figure 2:**
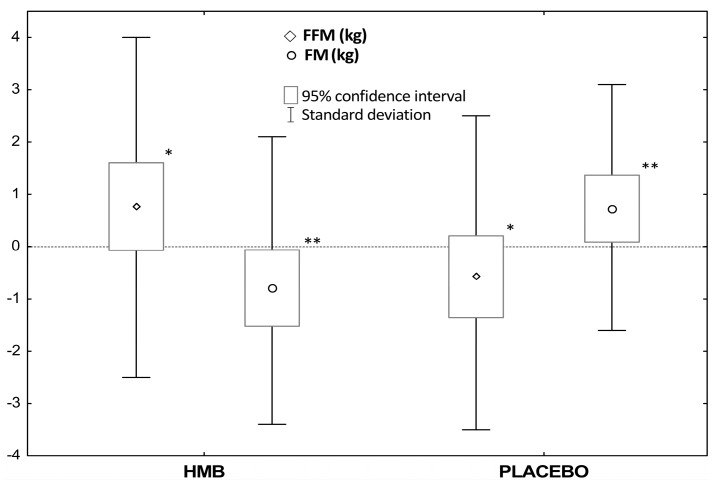
Changes in body composition after 12-week supplementation of HMB and placebo. Values are means ± SD and 95% CI. Abbreviations: HMB, β-hydroxy-β-methylbutyric acid; FFM, fat-free mass; FM, fat mass. Significant differences compared with placebo: * *p* = 0.049 (dependent samples *t*-tests), ** *p* = 0.016 (dependent samples *t*-tests).

Following the 12-week HMB supplementation of the athletes, in comparison to the placebo treatment, a significant increase (*p* = 0.049) in the fat-free mass (HMB: +0.8 kg vs. PLA: −0.6 kg) was recorded. A reduction (*p* = 0.016) in the fat mass was also observed (HMB: −0.8 kg vs. PLA: +0.7 kg) (Figure 2). However, there was no effect of the supplementation order (HMB➔PLA vs. PLA➔HMB) on the body composition of the study participants (Table 2).

Moreover, in relation to the pre-treatment value, after HMB supplementation a significant (*p* = 0.029) decrease in fat mass was recorded, whereas after placebo administration fat mass increased (*p* = 0.011) (Table 2).

### 3.2. Aerobic Capacity

**Figure 3:**
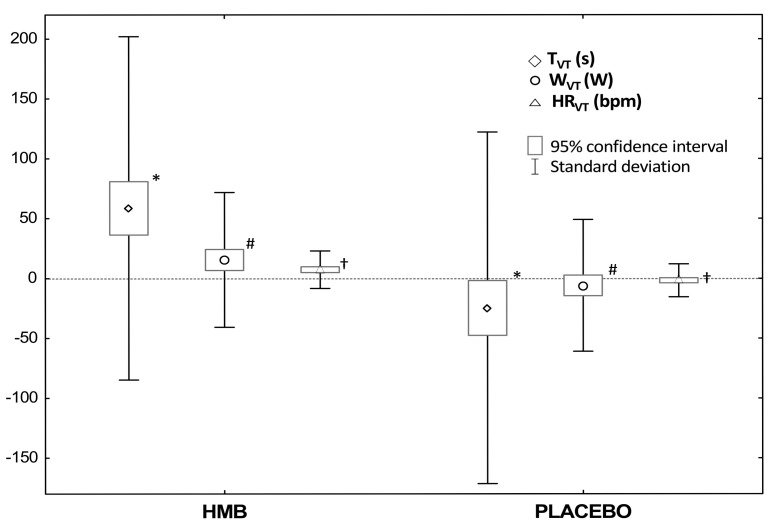
Changes in rates at ventilatory threshold after 12-week supplementation of HMB and placebo. Values are means ± SD and 95% CI. Abbreviations: HMB, β-hydroxy-β-methylbutyric acid; VT, ventilatory threshold; T_VT_, time to VT; W_VT_, load at VT; HR_VT_, heart rate at VT. Significant differences compared with placebo: * *p* < 0.0001 (dependent samples *t*-tests); # *p* = 0.017 (Wilcoxon rank-sum test); † *p* < 0.0001 (dependent samples *t*-tests).

The analysis of the aerobic capacity indices shows that, following HMB supplementation in comparison to the placebo administration, time to reach VT (T_VT_: +59 s_HMB_ vs. −25 s_PLA_, *p* < 0.0001), threshold load at VT (W_VT_: +15 W_HMB_ vs. −6 W_PLA_, *p* = 0.017) and the threshold HR at VT (HR_VT_: +7 bpm_HMB_ vs. −2 bpm_PLA_, *p* < 0.0001) increased significantly (Figure 3).

Moreover, in relation to the pre-investigation value, after HMB supplementation, a significant increase in Tref (+30 s_HMB_, *p* = 0.023), maximal load of cycloergometer (+13 W_HMB_, *p* = 0.04), maximal heart rate (+2 bpm_HMB_, *p* = 0.025), and T_VT_ (*p* < 0.0001), W_VT_ (*p* = 0.006) and HR_VT_ (*p* < 0.0001) were recorded (Table 3). In addition, after the placebo treatment, T_VT_ (*p* = 0.035) decreased. In addition, once again, there was no effect of the supplementation order (HMB➔PLA vs. PLA➔HMB) on the aerobic capacity of the examined athletes (Table 3).

### 3.3. Biochemical Blood Markers

An analysis of the changes in the tested biochemical markers in the blood of the athletes did not show any statistically significant differences between the HMB and the placebo treatment groups. Similarly, the order of HMB and PLA administration (HMB➔PLA vs. PLA➔HMB) had no influence on the biochemical markers concentration/activity of the examined group of athletes. However, after the placebo treatment, cortisol levels increased (+57 nmol/L, *p* = 0.009) and LDH activity decreased (−22 U/L, *p* = 0.013) in comparison to their initial concentration (Table 4).

### 3.4. Anaerobic Capacity

**Figure 4:**
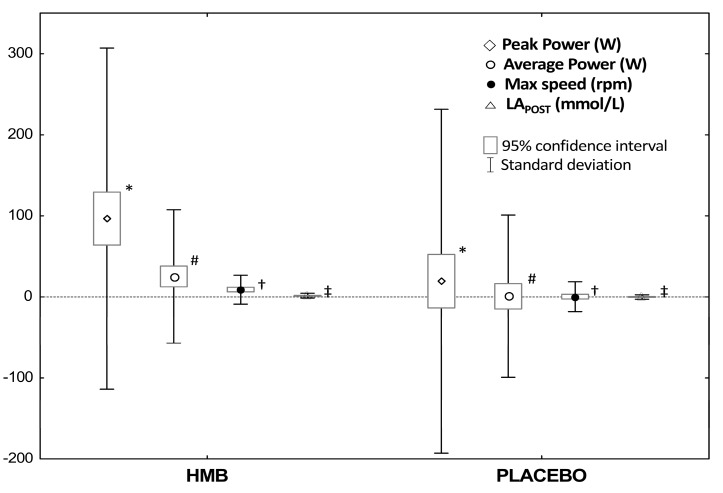
Changes in the anaerobic capacity after 12-week supplementation of HMB and placebo. Values are means ± SD and 95% CI. Abbreviations: HMB, β-hydroxy-β-methylbutyric acid; LA_POST_, post-exercise lactate concentrations. Significant differences compared with placebo: * *p* = 0.005 (dependent samples *t*-tests); # *p* = 0.029 (dependent samples *t*-tests); † *p* < 0.001 (dependent samples *t*-tests). ‡ *p* < 0.0001 (dependent samples *t*-tests).

Following the 12 weeks of supplementation with the HMB preparation, in comparison to the placebo treatment, a significant increase in the anaerobic peak power (+97 W_HMB_ vs. +19 W_PLA_, *p* < 0.001), average power (+25 W_HMB_ vs. +1 W_PLA_, *p* < 0.01), maximum speed (+9 rpm_HMB_ vs. 0 rpm_PLA_, *p* < 0.001), and post-exercise lactate concentrations (+1.6 mmol/L_HMB_ vs. −0.1 mmol/L_PLA_, *p* < 0.0001) was recorded (Figure 4).

Moreover, in relation to the pre-investigation value, after HMB supplementation, the peak power output (*p* < 0.0001), average power (*p* < 0.0001), maximum speed (*p* < 0.0001) and post-exercise lactate concentrations (*p* < 0.0001) increased, with a simultaneous reduction of the time needed to achieve peak power (*p* = 0.01) (Table 5). No significant differences were observed after the placebo treatment. The influence of the supplementation order (HMB➔PLA vs. PLA➔HMB) on the anaerobic capacity was also not shown.

## 4. Discussion

The findings of this study indicate that a 12-week HMB supplementation of athletes practicing combat sports is effective and, therefore, seems to be justified in such disciplines. It should also be stressed that the analysis of the obtained data proved that the supplementation order of the HMB and placebo treatment had no impact on the obtained results, which excludes the potential impact of the order on the registered parameters.

Our observations indicate that a 12-week HMB supplementation of athletes practicing combat sports results in a reduction of fat mass and an increase in the fat-free mass, without increasing the athletes’ body mass. This is particularly important in the sport disciplines with weight categories. The athletes training these sports should attempt to achieve and maintain a certain body mass, and regulate it mostly through lowering the amount of fat tissue, which can positively influence their physical performance, physical work capacity, and limit the adverse effects of rapid weight loss [60,61,62].

The study procedure applied in this research did not influence in any way the athletes’ lifestyle, training or diet. As mentioned in the methodological part, dietary and training recordings were made every second week, throughout the whole study, which indicated that athletes did not change their dietary habits or training characteristics during the HMB supplementation and the placebo period. The potential impact of these and other factors was also significantly reduced by the randomized crossover design used in the study. Although the aim of the study was not to improve the body composition, the lack of body mass changes in the athletes indicates that their energy supply provided by the diet covered the daily energy expenditure. Thus, it seems reasonable to conclude that HMB supplementation resulted in some desired changes in the body composition. It is worth mentioning that the authors of this manuscript are well aware of the potential limitations of the bioelectric impedance measuring method used in this study. However, the reliability of BIA methods depends on the strict observance and maintenance of the recommended measurement procedure, which is described in the Methods Section. Moreover, this body composition analysis method was also used in other research involving HMB supplementation [6,37].

In combat sports it is very often the ability to effectively attack or defend oneself with maximum strength, power and muscle speed that decides about the athlete’s final success, and these require a large anaerobic potential [61,62]. In our study, following the HMB supplementation, a significant increase in the anaerobic power and post-exercise lactate concentrations was recorded in comparison to the placebo treatment. This indicates that HMB positively supported anaerobic capacity and improved the athletes’ buffer capacity. Moreover, apart from the mentioned indices, after the HMB supplementation, there was also an increase in the maximum speed with a simultaneous reduction of time needed to achieve peak power in comparison to the pre-investigation value.

Therefore, it can be assumed that both in terms of the beneficial influence on the body composition regulation process and a significant anaerobic capacity stimulation, the supply of HMB seems to support the development of the potential efficiency of the supplemented athlete during a sports combat. The above hypothesis concerning the HMB influence on the body composition, or anaerobic capacity may be confirmed by the studies in which the individuals supplemented with HMB performed only resistance exercise stimulating (to a greater extent) an increase in fat-free body mass [2,35,36,38], muscle strength [2,3,36,37,38] and power [36,38] or a decrease of fat mass [3,36,37,38]. For example, in the study of Nissen et al. [2] three-week supplementation demonstrated an increase in FFM and total muscle strength. Similar results concerning an increase in muscle strength (leg extension) was observed by Thomson et al. [37], who for nine weeks supplemented male athletes experienced in resistance training. These authors also recorded a reduction in fat mass (−7%_HMB_ vs. +1%_PLA_). In turn, in experiments on volleyball players, where the key role was muscle power, upon HMB supplementation there was an increase in FFM (+2.3 kg_HMB_ vs. −0.1 kg_PLA_), peak (+1.7 W/kg_HMB_ vs. +0.4 W/kg_PLA_) and average power (+0.9 W/kg_HMB_ vs. +0.1 W/kg_PLA_), as well as fat mass reduction (−0.6 kg_HMB_ vs. +0.6 kg_PLA_) [3]. In addition, over the 12-week resistance-training when HMB free acid was supplemented, there was an increase of lean body mass (+11.0%_HMB_ vs. +3.1%_PLA_), quadriceps depth (+14.3%_HMB_ vs. +4.8%_PLA_), total strength (+18.1%_HMB_ vs. +5.9%_PLA_), Wingate peak power (+18.1%_HMB_ vs. +11.8%_PLA_) and vertical jump power (+19.0%_HMB_ vs. +12.1%_PLA_), as well as a decrease in FM (−30.2%_HMB_ vs. −9.7%_PLA_) [38]. However, it may be worth mentioning the controversy surrounding this manuscript (protocols, control groups and extraordinary level of homogeneity) [63].

Moreover, aerobic capacity and endurance may also play the key role in combat sports. They determine the athlete’s ability to sustain a longer combat with high intensity, and they also influence the work or combat capacity of the athletes, who have to go through a few strenuous fights in one day during a competition [60,61]. We need to stress here that there is a limited amount of literature assessing the effect of HMB intake in endurance training, which is also additionally done in combat sports (e.g., running or cycling). After a two-week HMB supplementation in cyclists, Vukovich and Dreifort [51] recorded an increase in peak oxygen uptake ($\dot{V}$O_2_peak), an extension of the time required to reach $\dot{V}$O_2_peak, an increase in the lactate threshold (%$\dot{V}$O_2_peak) and a delayed OBLA (observed at the oxygen uptake) by 4.0%, 3.6%, 8.6% and 9.1%, respectively. Moreover, these indices were also higher in comparison to the results recorded in groups administered leucine or a placebo. Similar results were observed in rowers, whose $\dot{V}$O_2_max (+4.0%_HMB_ vs. −1.4%_PLA_) and VT (T_VT_: +9.6%_HMB_ vs. −1.6%_PLA_; W_VT_: +13.0%_HMB_ vs. −1.7%_PLA_; HR_VT_: +5.7%_HMB_ vs. +0.6%_PLA_) increased after a 12-week HMB supplementation, in comparison to both the placebo treatment and the values before the supplementation [6]. These results seem to confirm the effect of HMB supplementation observed in our study: The increase in aerobic adaptation of athletes. Furthermore, the observations also correspond to the latest results reported by Robinson et al. [49], who in a group of males and females after a four-week HMB supplementation combined with high-intensity interval training found levels of $\dot{V}$O_2_peak higher by almost 5.9% and 9.8%, respectively, in comparison to the placebo and the control groups. The authors also found VT to be higher by almost 9.3% and 16.5%, respectively. Another important point was demonstrated by Lamboley et al. [48] in a previously described study. It was proved that HMB had advantageous effect on athletes as its supplementation resulted in a considerable increase in $\dot{V}$O_2_max by as much as 7.7 mL/kg/min. In both groups, a significant improvement was also found in VT (+11.1%_HMB_ vs. +9.0%_PLA_). Despite the increase in $\dot{V}$O_2_max values recorded in this study in the group supplemented with HMB, the differences were not high, which suggests that they may have resulted, to a considerable extent, from the fact that the study participants practiced sports as recreation and, prior to the onset of the experimental procedure, had no aerobic training. In contrast, our study involved trained combat sports athletes, in whose case even a slight increase in the aerobic adaptation may be considered a particularly advantageous factor that may contribute to improving their sport capacity.

In the case of combat sports and many other disciplines, especially those involving weight categories, it is also important that HMB may attenuate muscle loss and slow down the decrease in the level of strength, power and exercise capacity during the pre-competition body mass reduction. In studies on mice, in the case of a sustained energy deficit induced by calorie restriction and endurance exercise, Park et al. [28] observed that HMB supply slowed down the decrease in grip strength (−0.8%_HMB_), increased the gastrocnemius mass and myofiber cross-sectional area—they were, respectively, 10% and 35% higher than in the control group, whereas in the control group they significantly worsened. These observations would additionally bear out the hypotheses that HMB supplementation has a special effect in catabolic conditions. In the above studies, a greater lean body mass, sensorimotor function and strength in HMB vs. control group were observed under normal training conditions and with a proper ad libitum energy supply. In turn, in a study on judoists subjected to a three-day limitation of energy intake (20 kcal/kg_bm_/day), a reduction was recorded for fat mass (−0.85 percent point_HMB_ vs. +0.2 percent point_PLA_) only in the group of athletes supplemented with HMB, although no differences were found in the anaerobic power between athletes using HMB and a placebo [45]. Apart from the negative energy balance, this may have resulted from the fact that the HMB supplementation lasted only three days, which seems too short to cause significant changes in the systemic anaerobic potential. Studies of Towsend et al. [24] are also vital for combat sports and other disciplines as they indicate that HMB supplementation during an intensive training period (which is commonplace directly before a competition or during training camps) can increase the effectiveness of regeneration processes, owing to attenuated circulation of TNF-α, TNFR1 expression during the recovery and the initial immune response to intense exercise. What may also be of major significance in this case is the influence of HMB on the cell membrane integrity through de novo cholesterol synthesis [4,10,32].

It turns out that HMB supplementation applied in the study of Towsend et al. [24] had no effect on the activity of muscle damage markers. The data from publications also do not clearly show that HMB changes their concentration. Nissen et al. [2] and van Someren et al. [39] found a lower activity of CK and/or LDH in the blood of the examined individuals following HMB supplementation. In resistance-trained individuals during the overreaching cycle, HMB-FA attenuated the increase in CK activity (−2.3%_HMB_ vs. +108.2%_PLA_) [38]. The above observations seem to suggest that HMB supplementation may play a significant role in the reduction of muscle damage. However, long-term HMB supplementation in trained individuals, e.g., as a result of homeostatic mechanisms in the body, may reduce the influence of this substance on the body’s adaptation level, as verified by the analyses of the levels of standard biochemical markers in blood. To confirm this thesis, Gallagher et al. [35] showed a lower CK activity (by approximately 200 U/kg) 48 h after a series of resistance exercises in a group receiving HMB; however, this effect disappeared after a longer supplementation period. In turn, Knitter et al. [47] observed lower concentrations of CK and LDH in a group of runners supplemented with HMB immediately after they completed a 20 km race, as well as during three successive days after this effort. The cited studies seem to confirm the hypothesis that HMB supplementation results in the stimulation of sarcolemma integrity and inhibition of proteolytic activity of the ubiquitin-proteasome system. This may indicate HMB supplementation in sports is advisable because it reduces the rate of muscle damage caused by intensive exercise loads.

It is important to observe that a limited number of studies have analyzed the effect of HMB uptake on the systemic hormone metabolism. In comparison to the resting-state hormone concentrations recorded prior to the tests and following 12 weeks of HMB administration combined with power training, Kraemer et al. [36] showed a significant increase in the pre-exercise concentration of testosterone and a reduction of cortisol levels, which were not observed in the control group. In the supplemented group, the blood testosterone concentration increased considerably 15 min after the completion of the exercise, but after 30 min the level of this hormone was similar to that recorded in the control group. No significant differences were observed in the blood concentration of cortisol, although in the supplemented group, a reduced level of cortisol was found 30 minutes after the exercise. However, it should be noted that supplement given in the above study included more than HMB (one serving contained: HMB, arginine, glutamine, taurine, and dextrose), which may have influenced hormones concentrations. In contrast, Wilson and colleagues [38] observed a decrease in cortisol levels (−0.5%_HMB_ vs. +23.0%_PLA_) in the HMB-FA supplemented resistance-trained individuals during the overreaching cycle [38]. Moreover, in a recent paper of Townsend et al. [7], testosterone levels significantly increased immediately after exercise in comparison to the baseline, but also returned to the previous level after 30 min in resistance trained men supplemented with HMB. This might explain no significant results observed in our study. We would like to highlight here that numerous study results are consistent with the results of our study and do not confirm the effect of HMB on the activity of CK and LDH [6,35,42,44,46,52] or the blood testosterone and/or cortisol concentration [3,6,44,46] in comparison to the placebo. However, the above ambiguity concerning HMB supplementation may stem from the differences in the type of training and its influence on an athlete’s homeostasis, which may be the determining factor for the effectiveness of such a medication or supplement. In the case of rowers supplemented with HMB for 12 weeks, who mainly went through endurance training, there was an increase in $\dot{V}$O_2_max and FM reduction observed, but no changes in FFM and anaerobic capacity [6]. On the other hand, in the case of the mentioned volleyball players doing speed, power and resistance training there was an increase of power, strength and FFM observed, and a reduction of FM, with no changes of $\dot{V}$O_2_max [3]. It should be emphasised that the specific nature of combat sports imposes not only anaerobic and mixed exercises, but also some endurance exercises, which may explain the changes in both anaerobic and aerobic adaptation observed in the participants of this study.

The current study and the available publications seem to clearly suggest that the benefits of HMB supplementation may be observed, not just in the case of constant training volume, but especially when the muscular damage is heightened [5,38,44]. Thus, as put forward by Nosaka et al. [64], the training procedure should be diverse and progressive. Therefore, in highly-trained subjects, the exercise stimulus must be stronger than in the untrained ones to cause significant disruption and stimulate, among others, the synthesis of muscle proteins or suppress catabolic conditions. As postulated in literature, an appropriate load of the body by the training or exercise can be a condition necessary for HMB to take part on anabolic signaling in the activation of, for example, the MAPK/ERK, PI3K/Akt, and mTOR kinase pathways [9,11,23,29], insulin-like growth factor 1 (IGF-1) [7,29,30,31] and growth hormone (GH) [7,30,31] expression, as well as anti-catabolic action, such as downregulation of the autophagic-lysosomal pathway and reduction of ubiquitin-proteasome system activity [10,13,25,26,27]. In turn, as observed in our study, the fat mass changes seem to be explained by an increase in fatty acid oxidation, as well as lipolysis and insulin sensitivity (e.g., due to the stimulation of the activation of AMPK kinase, Sirt1 and the dependent metabolic pathways) [33]. The FM reduction observed in numerous studies can also result from lipolysis stimulation by the growth hormone although, in contrast to Towsend et al. [7], Portal et al. [3] did not observe any changes in GH concentration after HMB supplementation. This, however, may stem from different times of HMB intake in those studies and also differences in the type of exercise that the study participants did.

Considering the results of our study demonstrating that HMB supplementation improves aerobic capacity, as well as the publications mentioned above, the observed changes may stem from some potential mechanisms of HMB action, connected with, for instance, regulation of muscle protein expression, maintenance of cell wall integrity or stimulation of AMPK kinase and Sirt 1 activity, which promotes stimulation of mitochondrial biogenesis, higher oxygen consumption and increased efficiency of carbohydrate, glycogen and fat metabolism [33,34,65,66]. Moreover, HMB can be converted to beta-hydroxy methylbutyrate-CoA and then to HMG-CoA, which is a precursor in cholesterol synthesis or alternatively can be metabolized to acetyl-CoA, acetoacetyl-CoA, and ketone bodies (acetoacetate, 3-hydroxybutyrate, and acetone) [4,67,68,69]. Thus, in this pathway, HMB might not only be a precursor of sarcolemma stabilisation through de novo cholesterol synthesis, but also through acetyl-CoA or ketone bodies, it may serve as an invaluable energy substrate [4,67,68,69]. Ketone bodies serve as a fuel to the working muscle during endurance exercise and have a beneficial effect on athletic performance [70,71]. Considering our knowledge to date, this hypothesis seems to be borne out only by Pinheiro et al. [66] who studied rats supplemented with HMB, where a higher level of glycogen and ATP was found not only in fast-twitch muscles, but also in slow-twitch muscles. Therefore, if there are more of these energy sources, work and exercise capacity of the body might increase, in the case of both speed and strength training and endurance training. Hence, it may be inferred that HMB supplementation under specific conditions also seems to enhance the increase in physical capacity adaptation, not only through the mentioned protein synthesis stimulation and proteolysis suppression, but also through increasing the usability and availability of energy substrates. Some further research is necessary to verify and possibly confirm this hypothesis.

It should be mentioned here that when assessing the levels of biochemical markers following HMB supplementation, it is difficult to reliably compare the presented studies. The final results may have been affected not only by the mentioned different training stimuli, but also by the administered dose, or the timing and duration of supplementation [65]. The above observations concerning the relation between the duration of supplementation and the biochemical marker concentration (e.g., CK, testosterone or cortisol in blood) are inconclusive, while the results of some research contradict the postulates of our hypothesis [7,38,46]. The discrepancies in the results concerning HMB supplementation might not have resulted from the length of supplementation, but actually from the mentioned type of exercise stimulus (either appropriate or not strong enough). Therefore, HMB supplementation should be verified in the future on the basis of various controlled training programs carried out in both natural and laboratory conditions, which would take into account the changes and progression of the load and a proper muscle “disorientation”.

When assessing HMB supplementation in athletes, the type of the supplemented HMB should also be considered. Most of the available studies verified Ca-HMB supplementation [2,3,6,35,37,42,43,44,45,47,48,51,72]. Rather few studies used free acid form of HMB (HMB-FA), which proved to have a positive impact on lean body mass, muscle hypertrophy, strength, power, $\dot{V}$O_2_peak, and VT, as well as the level of the analyzed biochemical markers in blood (e.g., plasma growth hormone, IGF-1 (AUC), testosterone, cortisol, CK, TNF-α and TNFR1) [5,7,24,38,49]. The reason for this positive impact might be that absorption kinetics rather improve after HMB-FA intake than after Ca-HMB supplementation [73,74]. Moreover, in the last paper by Fuller et al. [74], HMB-FA in the capsule form was demonstrated to be characterized by a higher absorption efficiency, in comparison to the Ca-HMB. This indicates that future studies should consider supplementation of this HMB type.

The discrepancies in the results may also be caused by the fact that the dosing procedure is not clearly established. In the available literature, it is commonly accepted that the most recommended dose is about three grams of HMB per day [2,3,5,6,35,38,39,46,47,48,49,50,52,72]. The higher the dose, the higher the level of excreted HMB (at 1 g or 3 g of HMB, it amounts to, respectively, 14% and 29% of the amount of the administered dose) [53]. Nevertheless, Nissen et al. [2] observed that after a three-week supplementation of 1.5 g and 3 g of HMB, the lean body mass and muscle strength increased in proportion to the administered HMB amount. Gallagher et al. also provided the study participants with different HMB doses [35]. However, after supplementing 38 mg/kg_bm_/day (~3 g/day) and 72 mg/kg_bm_/day (~6 g/day) of HMB and the placebo, authors came up with inconclusive results. On the basis of the above mentioned works, it might be concluded that ~3 g of HMB is the appropriate amount though, as discussed, this could stem from the fact that the study participants were untrained and did only resistance exercise. Additionally, the training stimulus could be much more intensive for such athletes and stimulate to a large extent fat-free mass increase with the optimal dose of 3 g of HMB per day. However, it is possible that, in the case of trained athletes, if HMB is to be effective, not only some strengthening of the training stimulus would be required, but also an increase in the administered dose, which would be adequate to their relatively higher muscle mass or faster muscle metabolism.

Thus, it seems necessary to carry out in the future some studies on what the most beneficial dosing for trained athletes is. Moreover, such research should not only lead to establishing the recommended dose per day (as it has been to date), but also to developing an optimal method of calculating the HMB dose appropriate for the individual fat-free mass level in a given athlete. We would like to underline that in the future it would also be significant to establish the optimal time of day or the time before the training when HMB is to be administered. In the majority of the research to date, HMB was supplemented three times a day during a meal, but the timing was not relative to the training [2,5,39,42,43,44,45,47,48,51,52]. The studies where 3 g of HMB were consumed exclusively in the morning gave inconclusive results [3,46]. On the other hand, Robinson et al. [49], who administered HMB prior to exercise and again 1 hour later, and then 3 hours post exercise on training days, observed significant changes in $\dot{V}$O_2_peak and VT. In our study, HMB was administered upon waking, immediately after training, and before sleep. This type of procedure may explain the observed increase in anaerobic and aerobic exercise adaptation with a simultaneous lack of direct changes in hormone concentration or activity of enzymes analyzed after the training.

It could be suspected that HMB intake before exercise changes the concentration of those biochemical markers in blood. This hypothesis seems to be borne out by Towsend et al. [24], where HMB-FA in gel form was consumed 30 min before the exercise session, 2 h following the exercise session and 6 h following the exercise session. It was concluded that post-resistance exercise TNF-α and TNFR1 expression decreased. On the other hand, the intake of 1 g HMB-FA 30 min prior an acute heavy resistance exercise protocol resulted in a significant elevation of plasma GH (immediately after the exercise), AUC-IGF-1 and AUC-GH in the HMB group compared to the placebo group [7]. The research of Wilson et al. [72] is also worth mentioning in this context. These authors administered 3 g Ca-HMB an hour before 55 maximal eccentric knee extension or flexion contractions. In spite of the fact that there were no clear effects of HMB supplementation resulting from this acute dose or the timing, authors did observe some beneficial, though statistically insignificant, attenuation of: CK activity (at 48 h: HMB_3gPRE_: +324% vs. HMB_3gPOST_: +669% vs. CON: +535%) and LDH (at 72 h: HMB_3gPRE_: +56% vs. HMB_3gPOST_: +238% vs. CON: +229%), as well as an apparent decrease in quadriceps and hamstring soreness.

Considering the above, it seems feasible that in the future studies HMB supply should be coordinated with physical activity or training procedure. Therefore, the effective strategy might be HMB intake before and after the training, in the morning and before going to sleep.

## 5. Conclusions

This study indicates that HMB supplementation in athletes training combat sports promotes an advantageous reduction of fat mass and increase in fat-free mass, anaerobic peak power, average power, and post-anaerobic exercise lactate concentrations. HMB also stimulates beneficial changes in aerobic capacity of the organism, mainly due to the increased values of ventilatory threshold. Furthermore, long-term HMB supplementation does not seem to cause significant changes in creatine kinase and lactate dehydrogenase activity, or testosterone and cortisol concentration in blood.

## Figures and Tables

**Table 1:** Characteristics of the participating athletes (*N* = 42 subjects).

Variable	Unit	HMB	PLA	*p* Value ^a^ (HMB vs. PLA)
Age	(year)	22.8 ± 6.1		
Body mass	(kg)	81.2 ± 12.8		
Height	(cm)	179 ± 6		
Years training	(year)	7.5 ± 3.6		
Energy intake *	kcal	3081 ± 841	3141 ± 789	0.2965
	(kcal/kg/day)	37.7 ± 6.5	38.4 ± 5.6	0.1800
Protein intake *	g	141 ± 40	145 ± 44	0.1054
	(g/kg/day)	1.7 ± 0.3	1.8 ± 0.3	0.1181
	%	18.5 ± 3.5	18.6 ± 3.3	0.8175
Carbohydrate intake *	g	359 ± 112	374 ± 97	0.1683
	(g/kg/day)	4.4 ± 1.0	4.6 ± 0.8	0.0938
	%	46.6 ± 6.6	47.7 ± 5.2	0.1535
Fat intake *	g	120 ± 44	118 ± 41	0.3202
	(g/kg/day)	1.5 ± 0.4	1.4 ± 0.4	0.2135
	%	34.9 ± 7.2	33.7 ± 6.2	0.1109
Combat sports training *	(session/week)	5.2 ± 1.4	5.2 ± 1.3	0.8446
Supporting endurance training (running, cycling and other) *	(session/week)	1.3 ± 0.9	1.4 ± 0.8	0.3942
Supporting strength and power training *	(session/week)	1.9 ± 0.8	1.8 ± 0.7	0.4080
The total amount of all trainings *	(session/week)	8.3 ± 2.3	8.3 ± 2.1	0.8734

Values are means ± SD. Abbreviations: HMB, β-hydroxy-β-methylbutyric acid; PLA, placebo. * Data registered during the supplementation period. ^a^ Depending on the data distribution: dependent samples *t*-tests or Wilcoxon-signed rank tests.

**Table 2:** Body mass and body composition indices before and after the 12-week supplementation with HMB and placebo preparation.

Parameter		PRE: HMB vs. PLA	HMB	HMB: Pre vs. Post	PLACEBO	PLA: Pre vs. Post	POST: HMB vs. PLA	SO (HMB➔PLA vs. PLA➔HMB)
		*p* Value ^a^		*p* Value ^b^		*p* Value ^b^	*p* Value ^a^	*p* Value ^c^
Body composition indices								
Body mass (kg)	Pre	0.978	81.1 ± 12.7	0.905	81.0 ± 12.6	0.627	0.936	0.690
	Post		81.0 ± 12.2		81.2 ± 12.1			
TBW (L)	Pre	0.821	49.6 ± 6.1	0.079	49.9 ± 6.2	0.208	0.626	0.938
	Post		50.1 ± 5.8		49.5 ± 5.9			
FFM (kg)	Pre	0.817	67.7 ± 8.4	0.071	68.1 ± 8.4	0.146	0.599	0.937
	Post		68.5 ± 8.0		67.6 ± 8.0			
FFM (%)	Pre	0.558	84.0 ± 4.6	***0.014***	84.6 ± 5.0	***0.019***	0.213	0.146
	Post		85.0 ± 5.1		83.7 ± 4.6			
FM (kg)	Pre	0.639	13.4 ± 5.5	***0.029***	12.9 ± 5.8	***0.011***	0.334	0.317
	Post		12.6 ± 5.7		13.6 ± 5.4			
FM (%)	Pre	0.568	16.0 ± 4.6	***0.019***	15.4 ± 5.0	***0.020***	0.224	0.153
	Post		15.0 ± 5.0		16.3 ± 4.5			

Values are means ± SD. Abbreviations: HMB, β-hydroxy-β-methylbutyric acid; PLA, placebo, SO, supplementation order (HMB➔PLA vs. PLA➔HMB); TBW, total body water content; FFM, fat-free mass; FM, fat mass. ^a^ Depending on the data distribution: independent samples *t*-tests or Mann–Whitney U tests; ^b^ Depending on the data distribution: Dependent samples *t*-tests or Wilcoxon-signed rank tests; ^c^ ANOVA for repeated measurement.

**Table 3:** Levels of the monitored aerobic capacity indices before and after the 12-week supplementation with HMB and placebo preparation.

Parameter		PRE: HMB vs. PLA	HMB	HMB: Pre vs. Post	PLACEBO	PLA: Pre vs. Post	POST: HMB vs. PLA	SO (HMB➔PLA vs. PLA➔HMB)
		*p* Value ^a^		*p* Value ^b^		*p* Value ^b^	*p* Value ^a^	*p* Value ^c^
$\dot{V}$O_2_max (mL/min/kg)	Pre	0.596	57.3 ± 7.3	0.083	58.2 ± 7.5	0.455	0.498	0.724
	Post		58.6 ± 6.1		57.7 ± 6.8			
$\dot{V}$O_2_max (mL/min)	Pre	0.598	4603 ± 624	0.115	4663 ± 625	0.537	0.523	0.350
	Post		4709 ± 591		4627 ± 581			
Tref (s)	Pre	0.623	719 ± 114	***0.023***	746 ± 139	0.851	0.567	0.085
	Post		753 ± 140		751 ± 171			
Wmax (W)	Pre	0.452	281 ± 33	***0.040***	290 ± 42	0.856	0.870	0.060
	Post		294 ± 42		292 ± 48			
Wmax (W/kg)	Pre	0.326	3.5 ± 0.5	0.051	3.6 ± 0.5	0.951	0.971	0.293
	Post		3.7 ± 0.5		3.6 ± 0.6			
HRmax (bpm)	Pre	0.459	181 ± 9	***0.025***	182 ± 10	0.325	0.922	0.129
	Post		183 ± 10		183 ± 10			
T_VT_ (s)	Pre	0.074	505 ± 91	***<0.0001***	543 ± 100	***0.035***	***0.035***	0.080
	Post		564 ± 89		518 ± 105			
W_VT_ (W)	Pre	0.206	220 ± 29	***0.006***	231 ± 29	0.221	0.112	0.181
	Post		236 ± 28		225 ± 34			
W_VT_ (W/kg)	Pre	0.111	2.7 ± 0.4	***0.002***	2.9 ± 0.4	0.098	0.202	0.625
	Post		2.9 ± 0.4		2.8 ± 0.4			
HR_VT_ (bpm)	Pre	0.055	158 ± 10	***<0.0001***	163 ± 12	0.105	0.069	0.111
	Post		165 ± 11		161 ± 11			
	Post		13.1 ± 1.7		12.1 ± 1.4			

Values are means ± SD. Abbreviations: HMB, β-hydroxy-β-methylbutyric acid; PLA, placebo; SO, supplementation order (HMB➔PLA vs. PLA➔HMB); $\dot{V}$O_2_max, maximal oxygen uptake; Tref, exercise time before athlete’s refusal to continue exercising; Wmax, maximum load; HRmax, maximum heart rate; VT, ventilatory threshold; T_VT_, time to VT; W_VT_, load at VT; HR_VT_, heart rate at VT. ^a^ Depending on the data distribution: independent samples *t*-tests or Mann–Whitney U tests; ^b^ Depending on the data distribution: dependent samples *t*-tests or Wilcoxon-signed rank tests; ^c^ ANOVA for repeated measurement.

**Table 4:** Levels of the monitored selected biochemical markers before and after the 12-week supplementation with HMB and placebo preparation.

Parameter		PRE: HMB vs. PLA	HMB	HMB: Pre vs. Post	PLACEBO	PLA: Pre vs. Post	POST: HMB vs. PLA	SO (HMB➔PLA vs. PLA➔HMB)
		*p* Value ^a^		*p* Value ^b^		*p* Value ^b^	*p* Value ^a^	*p* Value ^c^
CK (U/L)	Pre	0.737	310 ± 275	0.778	268 ± 165	0.198	0.400	0.591
	Post		302 ± 226		258 ± 194			
LDH (U/L)	Pre	0.817	320 ± 57	0.903	323 ± 61	***0.013***	0.114	0.721
	Post		321 ± 63		301 ± 53			
Testosterone (nmol/L)	Pre	0.940	16.5 ± 5.4	0.053	16.5 ± 4.4	0.336	0.403	0.116
	Post		18.5 ± 7.9		17.1 ± 4.4			
Cortisol (nmol/L)	Pre	0.895	499 ± 154	0.063	495 ± 160	***0.009***	0.900	0.968
	Post		545 ± 170		552 ± 168			
T/C ratio (T/C·10)	Pre	0.546	3.68 ± 1.94	0.886	3.75 ± 1.81	0.138	0.567	0.232
	Post		3.68 ± 1.88		3.37 ± 1.30			
Lactate (mmol/L)	Pre	0.886	1.7 ± 0.6	0.180	1.7 ± 0.7	0.282	0.854	0.350
	Post		2.0 ± 1.1		1.9 ± 0.8			

Values are means ± SD. Abbreviations: HMB, β-hydroxy-β-methylbutyric acid; PLA, placebo; SO, supplementation order (HMB➔PLA vs. PLA➔HMB); CK, creatine kinase; LDH, lactate dehydrogenase; T/C ratio, testosterone/cortisol ratio. ^a^ Depending on the data distribution: independent samples *t*-tests or Mann–Whitney U tests; ^b^ Depending on the data distribution: dependent samples *t*-tests or Wilcoxon-signed rank tests; ^c^ ANOVA for repeated measurement.

**Table 5:** Levels of the monitored anaerobic capacity parameters before and after the 12-week supplementation with HMB and placebo preparation.

Parameter		PRE: HMB vs. PLA	HMB	HMB: Pre vs. Post	PLACEBO	PLA: Pre vs. Post	POST: HMB vs. PLA	SO (HMB➔PLA vs. PLA➔HMB)
		*p* Value ^a^		*p* Value ^b^		*p* Value ^b^	*p* Value ^a^	*p* Value ^c^
Peak Power (W)	Pre	0.511	901 ± 197	***<0.0001***	930 ± 208	0.240	0.251	0.468
	Post		998 ± 197		949 ± 184			
Peak Power (W/kg)	Pre	0.312	11.1 ± 1.6	***<0.0001***	11.5 ± 1.7	0.236	***0.045***	0.692
	Post		12.3 ± 1.6		11.7 ± 1.6			
Time at PP (s)	Pre	0.761	2.98 ± 2.01	***0.009***	2.71 ± 1.49	0.975	0.301	0.826
	Post		2.36 ± 1.06		2.67 ± 1.34			
Average Power (W)	Pre	0.618	628 ± 109	***<0.001***	640 ± 117	0.910	0.597	0.529
	Post		653 ± 108		641 ± 99			
Average Power (W/kg)	Pre	0.307	7.7 ± 0.7	***<0.0001***	7.9 ± 0.7	0.811	0.265	0.865
	Post		8.1 ± 0.6		7.9 ± 0.6			
Minimal Power (W)	Pre	0.758	379 ± 70	0.581	374 ± 78	0.435	0.917	0.614
	Post		385 ± 65		383 ± 61			
Minimal Power (W/kg)	Pre	0.591	4.7 ± 0.7	0.604	4.7 ± 0.9	0.846	0.822	0.813
	Post		4.8 ± 0.6		4.7 ± 0.6			
Max speed (rpm)	Pre	0.265	129 ± 14	***<0.0001***	132 ± 15	0.803	0.083	0.474
	Post		138 ± 13		133 ± 13			
Lactate_PRE_ (mmol/L)	Pre	0.664	1.6 ± 0.4	0.346	1.6 ± 0.5	0.153	0.851	0.150
	Post		1.5 ± 0.5		1.5 ± 0.6			
Lactate_POST_ (mmol/L)	Pre	0.078	11.6 ± 1.7	***<0.0001***	12.2 ± 1.6	0.482	***0.002***	0.978
	Post		13.1 ± 1.7		12.1 ± 1.4			

Values are means ± SD. Abbreviations: HMB, β-hydroxy-β-methylbutyric acid; PLA, placebo; SO, supplementation order (HMB➔PLA vs. PLA➔HMB). ^a^ Depending on the data distribution: independent samples *t*-tests or Mann–Whitney U tests; ^b^ Depending on the data distribution: Dependent samples *t*-tests or Wilcoxon-signed rank tests; ^c^ ANOVA for repeated measurement.

## Supplementary Materials

The following are available online at www.mdpi.com/2072-6643/9/7/753/s1, File S1: Study protocol approved by IRB, Table S1: CONSORT checklist.
