# Supplementary material for: The Effect of a 12-Week Beta-hydroxy-beta-methylbutyrate (HMB) Supplementation on Highly-Trained Combat Sports Athletes: A Randomised, Double-Blind, Placebo-Controlled Crossover Study
Source: Nutrients. 2017 Jul 14;9(7):753. doi: 10.3390/nu9070753 (PMC5537867; doi:10.3390/nu9070753)
Supplement: Supplementary file 1 [file nutrients-09-00753-s001.zip › S1 File. Study protocol approved by IRB.pdf]

Poznan University of Life Sciences
Faculty of Food Science and Nutrition
Department of Hygiene and Human Nutrition
Department Chair: Professor Jan Jeszka
Principal Investigator: Krzysztof Durkalec-Michalski
Wojska Polskiego 31, 60-637 Poznan, Poland
Phone/Fax: (+48 61) 848 73 32

STUDY PROTOCOL

THE EFFECT OF Β-HYDROXY-Β-METHYLBUTYRATE (HMB) SUPPLEMENTATION ON PHYSICAL CAPACITY AND BODY COMPOSITION IN TRAINED ATHLETES

1. Introduction

Adequate dietary supplementation may significantly affect human health and metabolism, as well as exercise capacity and physical capacity. However, it needs to be stressed, that the application of substances having physiological effects and nutritive value should only be a supplementation of healthy diet. It is also essential to use these preparations in justified cases and as evidence-based supplementation, such as supported by solid research findings concerning mechanisms of their action and the effect of these substances on the human body.

Among the numerous groups of supplements, beta-hydroxy beta-methylbutyric acid (HMB) is of particular interest, as it has been investigated for over 20 years in many studies, but it has not been conclusively stated the impact of this preparation over a longer period of time in trained athletes. In view of the inconclusive character of HMB research, the aim of this study was to verify the effect of HMB supplementation on body composition, aerobic and anaerobic capacity, and on the levels of biochemical markers in trained athletes, in randomised, placebo controlled, double-blind crossover trials.

2. Specific character of conducted supplementation

In this project supplementation will be conducted following the highest standard of randomized, placebo controlled, double blind test trials.
After randomization to specific groups athletes twice within 12 weeks will be supplemented in a split dose (one capsule upon waking, one capsule immediately after training, and one capsule before sleep, and on non-training days: one serving with each meal throughout the day) of a total of recommended intake of 3 g HMB per day or placebo (Gallagher et al., 2000; Hoffman et al., 2004; Lamboley et al. 2007; Nissen et al., 1996; Ransone et al., 2003; Van Someren et al., 2005; Vukovich & Dreifort 2001).

Supplementation periods will be separated by a 10-day washout period.

Among all participants, the efficacy of HMB supplementation will be assessed using four series of research (each included: evaluation of body composition, aerobic and anaerobic capacity, as well as blood sampling and biochemical analyses), consisting of identical procedures in two cycles separated by a washout period: the tests took place prior to the onset

of the intervention (Pre_{HMB} and Pre_{PLA}), following the 12 weeks of supplementation with the HMB preparation ($Post_{HMB}$) and the placebo ($Post_{PLA}$).

3. Characteristics of tested population

The research will be carried out with the participation of a group of trained athletes.
The study group will consist of about 60 athletes.

Inclusion Criteria:

- Gender: Male
- Minimum Age: 17 Years
- Maximum Age: 40 Years
- Trained sport discipline: combat sports and endurance sports,
- Training experience: at least 5 years,
- Minimum of 6 workout sessions a week (minimum 3 in the practiced sports discipline).
- Written consent to participate of study procedure,
- Currently issued medical certificate confirming their capacity to practice sports,

Exclusion Criteria:

- Failure to follow the study protocol,
- Injury during study protocol,
- Any health counter-indication,
- Failure to perform exercise procedures,
- Declared general feeling of being unwell.

4. Methods

4.1. Evaluation of anthropometric indices and body composition

4.1.1. Evaluation of body mass and height

Evaluation of body mass and height in the examined group of athletes will be conducted using medical scales certified for compliancy with the standard and medical directives, legalized and coupled with a WPT 60/150 OW anthropometer by RADWAG® (Polska). Analyses of body mass and height will be conducted in the morning using all recommended measurement procedures (CDC 2007).

4.1.2. Evaluation of body composition by electrical bioimpedance

Analysis of body composition by electrical bioimpedance will be conducted using a 4-electrode BIA 101S analyser by AKERN-RJL (Italy) and the Bodygram 1.31 computer software by AKERN-RJL (Italy).

Body composition will be measured strictly following the recommended measurement conditions, i.e. in the morning hours, following overnight fasting, in subjects lying in a supine position, and with the recommended application of measuring electrodes (Kyle et al., 2004;

Lukaski and Johnson 1986). Athletes will be also instructed to abstain from drinking coffee, strong tea, caffeine-containing products, and alcohol for at least 24 h before the test, as well as to refrain from physical exercise for a minimum of 18 h before measurements.

4.2. Evaluation of aerobic and anaerobic physical capacity

Exercise tests evaluating selected capacity indices of athletes will be conducted at the Laboratory of Exercise Tests, Department of Human Nutrition Hygiene, the Poznań University of Life Sciences, located in the building of the Faculty of Food Science and Nutrition. During the tests all required conditions recommended for such tests will be followed. Ambient temperature will be 20-22ºC, at relative humidity of approx. 50-60%. Tests will be conducted in the morning hours, constant for each participant, from 7:00 to 10:30 a.m.

4.2.1. Evaluation of aerobic capacity of athletes

Evaluation of aerobic capacity of athletes, based on e.g. the determination of their maximal oxygen uptake (VO_2max) and ventilatory threshold (VT), will be conducted using an increasing intensity exercise test on a Kettler X1 cycloergometer (Germany) following recommendations for such tests (Winter et al., 2009). The exercise test will be started at rotation frequency of 70±3 RPM/min and initial load of 100 W, which will be increased by 50 W at every 3 minutes. The exercise test will be conducted until the participant refuses to continue, it is impossible to maintain the set rotation frequency on the cycloergometer or a lack of increase in oxygen uptake, which will indicate that the individual maximal exercise load has been reached.

During the exercise tests physiological and metabolic indices will be recorded using a portable $K4b^2$ ergospirometer (Cosmed, Italy), and COSMED CPET Software Suite.

4.2.1.1. Determination of maximal oxygen uptake

Determination of maximal oxygen uptake during the exercise test with an increasing load will be conducted based on the analysis of changes in oxygen uptake, depending on the intensity of physical exercise. The recorded measurement of oxygen uptake in that apparatus will be based on the standard method proposed by Douglas (Bruce et al., 1963). Reaching maximal exercise will be interpreted as the moment of a lack of increment in oxygen uptake (VO_2) and heart rate (HR) and/or the participant's refusal to continue exercise.

4.2.1.2. Determination of ventilatory threshold

Determination of ventilatory threshold as a marker of the anaerobic metabolism threshold will be conducted by the *V-slope* method, based on the analysis of linear regression of the curve of increasing CO_2 release in relation to the curve of increasing O_2 uptake and the determination of a point, at which the straight linear course of the dependence between VCO_2 and VO_2, observed from the beginning of the test, starts to intensively increase due to a disproportional increment in the exhaled CO_2 in relation to O_2 uptake (Beaver et al., 1986).

The ventilatory threshold (VT) determined during the increasing intensity exercise test will be characterized based on such indices as time to reach ventilatory threshold (T_{VT}), threshold load (W_{VT}) and threshold heart rate (HR_{VT}).

4.2.2. Determination of anaerobic capacity

Anaerobic capacity will be assessed using the classical Wingate test on a cycloergometer (Monark 894E, Sweden) following recommendations for such tests proposed by Bar-Or (1987).

Seat height will be adjusted to each participant's satisfaction, and toe clips with straps will be used to prevent the feet from slipping off the pedals.

The primary test will be preceded by a 5-min warm-up period of approximately 50 W power, followed by a 5-min break. The test will last for 30 s. External loading will be estimated individually at 7.5 % body weight. During the test, the athletes will be encouraged to maintain maximum effort. Recorded results included the peak power output, the average power output, the mean power output (MP), and maximum speed which will be analysed using Monark Anaerobic Test Software (ver. 3.0.1, 2009, Sweden).

4.3. Biochemical analyses of selected blood markers

The most widely used markers of adaptation and homeostasis in studies involving athletes will be applied in this investigation. The activity of the creatine kinase (CK) and lactate dehydrogenase (LDH) enzymes, and the concentration of testosterone and cortisol will be assessed based on a quantitative analysis of the blood plasma of the athletes using commercial diagnostic tests.

Twenty to twenty-five minutes after the incremental aerobic exercise test, blood samples will be collected from the athletes from the ulnar vein to two closed-system evacuated test tubes of 2.7 mL, using lithium heparine and sodium fluoride as anticoagulants (Sarstedt Monovette®, Germany). The collected plasma will be subjected to further laboratory analyses on the same day. CK and LDH activity, and the lactate concentration will be assayed using a standardised colorimetric enzymatic method with a COBAS® 6000 analyser (module c 501, Roche/Hitachi, USA). The concentrations of testosterone and cortisol in blood plasma will be assayed by ECLIA electrochemiluminescence using a COBAS® 6000 analyser (module e 601, Roche/Hitachi, USA).

Moreover, in this research at rest and three minutes after the Wingate anaerobic test, lactate concentration in capillary blood will be determined using the spectrophotometric enzymatic method (Maughan 1982).

4.4. The methodology of biochemical assays in urine

Analysis of hydration (urine specific gravity) will be made by osmometer osmometer 800CLG (Trident, Poland).

4.5. Evaluation of nutrition

Evaluation of nutrition will be conducted by continuous recording of all consumed foodstuffs, dishes and beverages, specifying home measures and weight (using "The Album of photographs of products and dishes"). Data will be analysed using the Dietetyk computer program (JuMar, Polska). Continuous recording of consumed food will cover every second week, throughout the whole study.

4.6. Evaluation of physical activity

During HMB and placebo supplementation, the participants will be obliged to monitor their physical activity. For this purpose the method of activity recording will be applied, including records of the type and specific character of the training and activity, its duration, intensity and frequency.

5. Selected literature references

1. Bar-Or O. The Wingate anaerobic test: an update on methodology, reliability and validity. Sports Med. 1987;4: 381-394.
2. Beaver WL, Wasserman K, Whipp BJ. A new method for detecting the anaerobic threshold by gas exchange. J Appl Physiol., 1986, 60(6), 2020-2027.
3. Bruce RA, Blackmon JR, Jones JW, Strait G. Exercise testing in adult normal subjects and cardiac patients. Pediatrics, 1963, 32(Suppl.), 742-756.
4. CDC - Centers for Disease Control and Prevention: National Health and Nutrition Examination Survey (NHANES). Anthropometry procedures manual, 2007. www.cdc.gov/nchs/data/nhanes/nhanes_07_08/manual_an.pdf
5. Gallagher PM, Carrithers JA, Godard MP, Schulze KE, Trappe SW. Beta-hydroxy-beta-methylbutyrate ingestion, Part I: Effects on strength and fat free mass. Med Sci Sports Exerc., 2000;32: 2109-2115.
6. Hoffman JR, Cooper J, Wendell M, Im J, Kang J. Effects of beta-hydroxy beta-methylbutyrate on power performance and indices of muscle damage and stress during high-intensity training. J Strength Cond Res., 2004, 18, 747-752.
7. Kyle UG, Bosaeus I, De Lorenzo AD, Deurenberg P, Elia M, Gómez JM, et al. Bioelectrical impedance analysis-part I: review of principles and methods. Clin Nutr., 2004, 23(5), 1226-1243.
8. Lamboley CR, Royer D, Dionne IJ. Effects of beta-hydroxy-beta-methylbutyrate on aerobic-performance components and body composition in college students. Int J Sport Nutr Exerc Metab. 2007;17: 56-69. doi: 10.1123/ijsnem.17.1.56
9. Livingstone MB, Robson PJ, Totton M. Energy expenditure by heart rate in children: an evaluation of calibration techniques. Med Sci Sports Exerc., 2000, 32(8), 1513-1539.
10. Lukaski HC, Johnson PC. Assessment of fat-free mass using bio-electrical impedance measurement of the human body. Am J Clin Nutr., 1985, 41(4), 810-817.
11. Maughan RJ. A simple, rapid method for the determination of glucose, lactate, pyruvate, alanine, 3-hydroxybutyrate and acetoacetate on a single 20-mul blood sample. Clin Chim Acta. 1982;122: 231-240.
12. Nissen S, Sharp R, Ray M, Rathmacher JA, Rice D, Fuller JC Jr, et al. Effect of leucine metabolite beta-hydroxy-beta-methylbutyrate on muscle metabolism during resistance-exercise training. J Appl Physiol., 1996, 81 (5), 2095-2104.
13. Ransone J, Neighbors K, Lefavi R, Chromiak J. The effect of beta-hydroxy beta-methylbutyrate on muscular strength and body composition in collegiate football
14. Van Someren KA, Edwards AJ, Howatson G. Supplementation with beta-hydroxy-beta-methylbutyrate (HMB) and alpha-ketoisocaproic acid (KIC) reduces signs and symptoms of exercise-induced muscle damage in man. Int J Sport Nutr Exerc Metab., 2005,15, 413-424.
15. Vukovich MD, Dreifort GD. Effect of beta-hydroxy beta-methylbutyrate on the onset of blood lactate accumulation and V(O)(2) peak in endurance-trained cyclists. J Strength Cond Res., 2001, 15, 491-497.

16. Winter EM, Jones AM, Davison RCR, Bromley PD, Mercer T. Sport and exercise physiology testing guidelines: vol. 1 Sport testing: The British Association of Sport and Exercise Sciences. Taylor and Francis e-Library, Abingdon 2009.
